# Perinatal Western Diet Consumption Leads to Profound Plasticity and GABAergic Phenotype Changes within Hypothalamus and Reward Pathway from Birth to Sexual Maturity in Rat

**DOI:** 10.3389/fendo.2017.00216

**Published:** 2017-08-29

**Authors:** Julie Paradis, Pierre Boureau, Thomas Moyon, Sophie Nicklaus, Patricia Parnet, Vincent Paillé

**Affiliations:** ^1^UMR 1280 Physiologie des Adaptations Nutritionnelles (PhAN), INRA, Université de Nantes, Institut des Maladies de l’Appareil Digestif (IMAD), Nantes, France; ^2^UMR 1324 Centre des Sciences du Goût et de l’Alimentation (CSGA), INRA, CNRS, Université de Bourgogne, Dijon, France

**Keywords:** reward, DOHaD, food preferences, nutrition, γ-aminobutyric acid, TaqMan low-density array

## Abstract

Perinatal maternal consumption of energy dense food increases the risk of obesity in children. This is associated with an overconsumption of palatable food that is consumed for its hedonic property. The underlying mechanism that links perinatal maternal diet and offspring preference for fat is still poorly understood. In this study, we aim at studying the influence of maternal high-fat/high-sugar diet feeding [western diet (WD)] during gestation and lactation on the reward pathways controlling feeding in the rat offspring from birth to sexual maturity. We performed a longitudinal follow-up of WD and Control offspring at three critical time periods (childhood, adolescence, and adulthood) and focus on investigating the influence of perinatal exposure to palatable diet on (i) fat preference, (ii) gene expression profile, and (iii) neuroanatomical/architectural changes of the mesolimbic dopaminergic networks. We showed that WD feeding restricted to the perinatal period has a clear long-lasting influence on the organization of homeostatic and hedonic brain circuits but not on fat preference. We demonstrated a period specific evolution of the preference for fat that we correlated with specific brain molecular signatures. In offspring from WD fed dams, we observed during childhood the existence of fat preference associated with a higher expression of key gene involved in the dopamine (DA) systems; at adolescence, a high-fat preference for both groups, progressively reduced during the 3 days test for the WD group and associated with a reduced expression of key gene involved in the DA systems for the WD group that could suggest a compensatory mechanism to protect them from further high-fat exposure; and finally at adulthood, a preference for fat that was identical to control rats but associated with profound modification in key genes involved in the γ-aminobutyric acid network, serotonin receptors, and polysialic acid–NCAM-dependent remodeling of the hypothalamus. Altogether, these data reveal that maternal WD, restricted to the perinatal period, has no sustained impact on energy homeostasis and fat preference later in life even though a strong remodeling of the hypothalamic homeostatic and reward pathway involved in eating behavior occurred. Further functional experiments would be needed to understand the relevance of these circuits remodeling.

## Introduction

Early life environment and events are now well recognized to contribute to health and disease predisposition later in life (1–3). The concept of metabolic imprinting has been proposed to describe how changes in the nutritional and hormonal environment during perinatal period can predispose the offspring to obesity and its associated pathologies later on. A significant issue of our occidental way of life is overnutrition as a consequence of the consumption of energy dense food. Indeed, individuals who are exposed to maternal intake of this type of food are at higher risk to develop obesity and metabolic syndrome (4, 5). Many studies have shown that maternal high-fat diet (HFD) through gestation and suckling has a long-term effect on offspring metabolism (6–8). In addition to pathways implicated in metabolic regulation, brain reward systems also play an important role in feeding behavior (9, 10). Mesolimbic dopamine (DA) neurotransmission, intensively studied in the context of reward and addiction, is altered in diet-induced obesity in both humans (11–13) and animals (14–16). DA projections develop, for a large part, postnatally (17), and therefore their development may be affected by early diet. Over the past few years, experiments on rodents evidenced that maternal HFD intake enhance hedonic feeding in offspring (18, 19). Even though this observation involved some changes in the DA system function (20–22), limited data are available concerning the ontogeny and the remodeling of the reward pathways during early life (21). Moreover whether and how the non-DA signaling part of the reward system such as the GABA (γ-aminobutyric acid) system could be affected by the perinatal nutritional stress is not documented. Indeed, GABA neurons seem to play a key role in reward and aversion. Ventral tegmental area (VTA) GABA neurons receive similar pattern of input from different brain areas (23), and recent optogenetic-based behavioral studies highlight the major role of VTA GABA in conditioned place aversion (24) and in reward consummatory behavior (25). Nucleus accumbens (NAc) is mainly constituted by the projection of the GABAergic medium spiny neurons and acts as a limbic–motor interface integrating signals arising from the limbic system and turning them into action *via* output to the ventral pallidum (VP) and others motor effectors (26). And finally, the hypothalamus that is constituted by numerous GABA connections in LH (27) and arcuate nucleus, integrates signals of hunger and satiety (10).

This study aims at identifying the influence of maternal western diet (WD) intake in the rat offspring from birth to sexual maturity (i) on fat preference (ii) on gene expression profile of the DA system, the GABAergic system and the plasticity of hypothalamus, and (iii) on the neuroanatomical/architectural changes of the mesolimbic dopaminergic networks for the same period. We therefore assessed, on a longitudinal study (from weaning, P25, to sexual maturity, P45 and adulthood, P95), the effect of maternal WD on body weight growth and adipose tissue development of offspring kept under regular chow after weaning. Concomitantly, we performed a fat preference test followed by a dedicated transcriptomic analysis and subsequent principal component analysis (PCA) of a selection of markers for food intake, choice and motivation regulatory systems. Our results significantly enriched the recent results focusing on nutritional programming of the DA system.

## Materials and Methods

### Ethics Statement

All experiments were performed in accordance with the guidelines of the local animal welfare committee, the EU (directive 2010/63/EU), the Institut National de la Recherche Agronomique (Paris, France), and the French Veterinary Department (A44276). The experimental protocol was approved by the institutional ethic committee and registered under reference APAFIS 8666. Every precaution was taken to minimize stress and the number of animals used in each series of experiments.

### Animals and Diets

**Figure 1:**
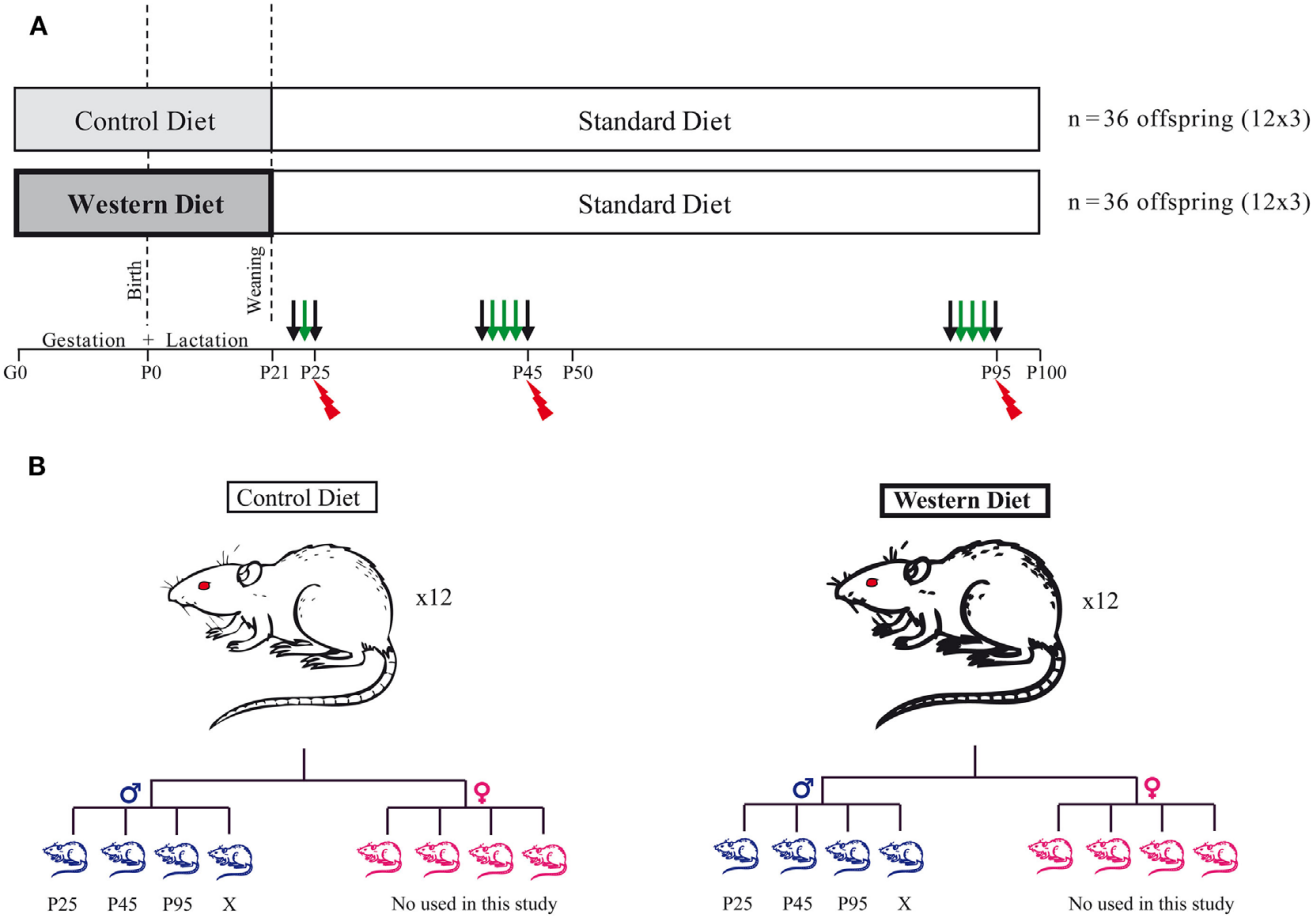
Experimental design. **(A)** Schematic diagram of the study design. Thirty-two female SPD rats at gestation day 1 (G1) were fed either a control diet for 16 of them or a western diet for the others during gestation and lactation period. At weaning, the offspring were all kept onto standard chow diet until the end of the experiment. At three different time points, P25 (childhood), P45 (Adolescence), and P95 (young adult), a fat preference test was performed for 1 day (P25) or 3 days (P45 and P95). *N* = 12 per group. The black arrows correspond to free access to two bottles with water, and the green arrows correspond to the 24 h fat preference test. Following the preference test, the rats were sacrificed (red spark). Half of them (*n* = 6 per group/age) were perfused by 4% paraformaldehyde and the brain removed for subsequent immunohistochemistry. The other half (*n* = 6 per group/age) was used for plasma measures and molecular analysis. **(B)** At birth, we kept 12 dams per diet, and we adjusted litter size to 8 pups/litter with a 1:1 male to female ratio. Only the males were then used in this study. For each time point (P25, P45, and P95), one rat per litter was used for behavior and subsequent postmortem analysis.

Animal were maintained in a 12 h/12 h light/dark cycle in a 22 ± 2°C with food and water *ad libitum*. Thirty-two female Sprague-Dawley rats (bodyweight: 240–290 g) at gestation day 1 (G1) were purchased directly from Janvier (Le Genest Saint Isle, France). They were housed individually and fed either a control diet (CD) (5% beef fat and 0% sucrose) for 16 of them or a WD (21% beef fat and 30% sucrose) for 16 of them during the gestation and lactation periods (see Table 1: diet composition in percent kcal from ABdiet Woerden, The Netherlands). At birth, litter size was adjusted to eight pups per litter with a 1:1 male to female ratio. We kept 12 out of 16 dams with a litter composed of 4 males and 4 females for each group. At weaning (P21), the offspring born to CD and WD dams were kept onto standard chow until the end of the experiment (Figures 1A,B). Pup body weight was recorded at birth and thereafter every day at 10:00 a.m. until P21 (weaning). After weaning and until the end of the experiment, rats were weighted every 3 days. We present data on male offspring only. Female rats were used for another study (Figure 1).

**Table 1:** Diet composition in percent kcal from each component of the maternal diets administered during gestation and lactation and standard diet for offspring.

Nutriments	Control diet	Western diet	Standard diet
Cellulose	4.22	5.0	3.9
Fat	5.06	21.0	3.1
Sucrose	0.0	29.45	2.0
Casein	18.55	22.0	16.1
Dextrose	8.43	10.0	12.2
Starch	57.5	5.0	45.8
Total energy, kcal/100 g	377.1	466.3	290

### Behavior (Two-Bottle Choice Test)

Three critical developmental periods were studied (P21 to P25: juvenile, P41 to P45: adolescence and P91 to P95: young adult). 24 male pups (*n* = 12 per group) were randomly selected and placed in an individual cage to perform a two-bottle choice free test (Figures 1A,B) (28–30). This test was used to specifically study the attractiveness toward fat taste by dissociating it from the sweet taste and as much as possible from the metabolic effect of calories intake. Indeed, 1% corn oil solution consumption is associated with an intake of 0.09 kcal/ml only. After one day of habituation to the presence of two bottles, the test was carried out over 2 days at P25 and over 4 days at P41 and P91 (Figure 1A). In details, at weaning (P21), 24 pups were housed individually for 2 days (Figure 1A): day 1, habituation phase, day 2, rats were given a two-bottle free choice between an emulsion of 1% corn oil in 0.3% xanthan gum (Sigma Aldrich, St. Quentin Fallavier, France) and xanthan gum solution (0.3%). At P41 and P91, 24 pups were used and two-bottle free choice was proposed for three consecutive days. The consumption of xanthan gum solution and taste solution (corn oil 1%) was recorded daily at 11:00 a.m. for 3 days (P45 and P95). The position of the two bottles was daily inverted to prevent position preference bias. The fat preference score was calculated as the ratio of “fat solution” volume consumed to the total volume consumed in 24 h. All rats were maintained under standard chow diet throughout the behavioral test.

### Tissues Collection and Blood Sampling

The day after the last day of the two-bottle free choice test, half of the rats (*n* = 6 per group) were rapidly euthanized between 09:00 and 12:00 a.m. by CO_2_ inhalation. Blood was collected in tubes with EDTA (Laboratoires Léo SA, St Quentin en Yvelines, France) and centrifuged at 2,500 *g* for 15 min at 4°C. Plasma was frozen at −20°C. Organs and individual retroperitoneal fat depot were dissected and weighted. The brain was rapidly removed and placed in a brain matrix (WPI, Sarasota, FL, USA rat 300–600 g). First the hypothalamus was dissected [according to Paxinos’s atlas coordinates: −1.0 to −4.5 mm from Bregma (31)] then, for each rat, two coronal slices of 2 mm thickness at the level of NAc and another one at the level of the VTA were obtained. Samples of the right and the left NAc and the right and the left VTA (four samples in total per animal) were rapidly obtained using two different biopsy punches (Stiefel Laboratories, Nanterre, France) (diameter of 4 mm for the NAc and 3 mm for the ventral midbrain). The samples were snapped frozen in liquid nitrogen and stored at −80°C for subsequent determination of gene expression by TaqMan low-density array (TLDA).

The other rats (*n* = 6 per group) were deeply anesthetized with pentobarbital (150 mg/kg i.p.) and perfused with a transcardial physiological saline perfusion followed by ice-cold 4% paraformaldehyde in phosphate buffer (PB), pH 7.4. The brains were rapidly removed, immersed in the same fixative for 1 h at 4°C, and finally stored in 25% PB sucrose for 24–48 h. The brains were then frozen in isopentane at −60°C, and finally stored at −80°C until use. The NAc, hypothalamus and VTA were cut into 20 µm serial coronal sections with a cryostat (Microm, Microtech, Francheville, France). Two or three series of 10 glass slides containing 4–6 sections were performed for each brain area. For each glass slide the serial sections are spaced of 200 µm (Figure 6).

### Biochemical Plasma Analyses

EDTA plasma collected on P25, P45, and P95 rats were used to measure plasma glucose, NEFA (non-esterified fatty acids), insulin, and leptin. Glucose and NEFA were measured using colorimetric enzymatic reactions with specific kits (glucose and NEFA PAP 150 kits, BioMérieux, Marcy-l’Etoile, France). Hormones were assayed with specific ELISA kits following the manufacturer’s instructions for insulin and leptin (rat/mouse insulin ELISA kit, rat leptin ELISA kit, Linco Research, St. Charles, MO, USA).

### Immunohistochemistry

Glass slides containing serial VTA and NAc sections were first blocked for 3–4 h and then incubated overnight at 4°C with a mixture of the following antibodies: mouse anti-NeuN (1:500; IgM; Millipore Bioscience Research Reagents, Merk, USA) and rabbit anti-TH (1:1,000; Millipore Bioscience Research Reagents, Merk, USA). After incubation with primary antibodies and subsequent washing with PB, sections were incubated in a mixture of secondary antibodies: Alexa 488 conjugated donkey anti-mouse IgM and Alexa 568-conjugated donkey anti-rabbit IgG (1:500; Invitrogen, ThermoFisher Scientific, Waltham, MA, USA) for 2 h. Sections were mounted in superfrost plus gold slides (ThermoFisher Scientific, Waltham, MA, USA), air-dried, and coverslipped with ProLong™ Gold antifade reagent (Invitrogen, ThermoFisher Scientific, Waltham, MA, USA).

#### TH Neurons Count in VTA

For each rat, TH-positive cells were counted as previously described (32) at three different rostrocaudal levels of the VTA: at the level of the exit of the third nerve (distance relative to Bregma: −5.3 mm), 200 µm rostral and 200 µm caudal to this level (Figures 6A). For the left and the right side, a digitized picture comprising the whole VTA from the accessory terminal tract medially to the lateral border of the mesencephalon was obtained using ×40 magnification of a NanoZoomer-XR Digital slide scanner C12000 (Hamamatsu, Japan). A line was drawn around the perimeter of the VTA for each section. The boundaries were chosen by examining the shape of the cells and referring to the Paxinos and Watson atlas. A dopaminergic neuron was defined as a NeuN(+)/TH(+) immunoreactive cell body with clearly visible nucleus. Using the NIH Image J software (cell counter plugin), the NeuN(+)/TH(+) cells were counted by two different persons with no knowledge of the animal groups. Split-cell counting errors were corrected using the formula of Abercrombie (32), where *N* = *n*[*t*/(*t* + *d*)] (*N* = total number of cells; *n* = number of cells counted; *t* = section thickness; and *d* = cell diameter), and this correction factor was 0.65. Data are expressed as mean [NeuN(+)/TH(+) in left and right VTA] ± SEM.

#### TH Fiber Density in NAc

The TH protein content in the dopaminergic nerve terminals of the NAc was estimated by anatomical densitometric analysis of TH immunolabeled sections. The TH fibers density was quantified at three arbitrary levels along the rostrocaudal axis of the NAc (Bregma 2.20, 1.70, and 1.20 mm) (Figure 6B). Briefly, digitized picture comprising the whole striatum and NAc obtained using ×40 magnification of a NanoZoomer-XR Digital slide scanner C12000 (Hamamatsu, Japan) were obtained. For a given NAc, a line was drawn around the whole nucleus to define the area of optical density (OD) measurement (Figure 6B). The obtained value was normalized with the OD value measured from a circular zone drawn on the corpus callosum (a region not stained for TH immunochemistry) of the same section using NIH Image J software. Data are expressed as a mean of the OD ratio (OD value in NAc/OD value in corpus callosum of the three sections) ± SEM.

### Gene Expression by TLDA and TaqMan

RNA was isolated from snap-frozen NAc, VTA-enriched samples, and hypothalamus, using the NucleoSpin RNA/protein kit (Macherey-Nagel, Hoerdt, France). Total RNA was submitted to DNase digestion following the manufacturer’s instructions, the quantity was estimated by the 260/280 nm UV absorbance, and the quality was assessed using the Agilent 2100 Bioanalyzer System, the RNA integrity number (RIN) was then calculated. Samples with a RIN below 8 were discarded. One microgram of total RNA was reverse transcribed into cDNA using High capacity RT kit (Applied Biosystems, Foster City, CA, USA) in a total volume of 10 µl.

As previously described (33), the TLDA is a 384-well micro-fluidic card on which 384 simultaneous real-time PCRs can be performed (Applied Biosystems, Foster City, CA, USA). We used a specifically designed TLDA made to cover different gene families relevant to plasticity and regulation of food intake. Each custom card was configured as 2 × 4-sample loading lines containing 2 × 48 reaction chambers (reference: 96a). A 92-gene set (Table S1 in Supplementary Material) and four housekeeping genes (18S, Gapdh, Polr2a, and Ppia) were studied. Real-time PCR was carried out using Life Technologies TaqMan reagents and run on ABI Prism 7900HT sequence detection system (Applied Biosystems, Foster City, CA, USA). Raw fluorescence data were collected through the PCR using the SDS 2.3 software (Applied Biosystems, Foster City, CA, USA), which further generated threshold cycles Ct with automatic determination of both baseline and threshold. After filtering using ThermoFisher cloud App (ThermoFisher, USA) to discriminate aberrant PCR runs, the assays per sample were *n* = 6 (*n* = 5 for WD group at P25). The data were then analyzed with ThermoFisher Cloud App (ThermoFisher, USA) for relative quantitation. Relative quantitation of gene expression (RQ) was based on the comparative Ct method using the equation RQ = 2^−ΔΔCt^, where ΔΔCt for one gene target was its own Ct variation subtracted from a calibrator sample and normalized with an endogenous control. Precisely, we determined the most stable housekeeping gene using geNorm algorithm (ThermoFisher Cloud App RQ, ThermoFisher, USA). Among the four housekeeping genes, Gapdh was defined as the endogenous control for NAc and hypothalamus, and Ppia for VTA and this was true for all samples from the three time periods analyzed. Graphic representation of genes expression was manually designed to assign one color for a 10% increment of gene expression relative to the CD group. Significant variation, using non-parametric Wilcoxon signed-rank test, was noted with an asterisk.

### Statistical Analysis

Results are expressed as mean ± SEM in tables and figures. Mann–Whitney non-parametric test was used for the analysis of Body weight at different time points, Fat preferences, and OD ratio obtained from the immunohistochemistry.

To assess the significance of the 3 days fat preferences, we performed a column statistic analysis for each day. For each group, consumption of fat solution and control solution was tested using the non-parametric Wilcoxon’s signed-rank test. We compared the preference mean value with the hypothetical value of 50% (dotted red line). Significant variation was noted with a red asterisk. We used the same test for the qPCR RQ value analysis; we compared the mean RQ value with the hypothetical value of 1. Significant variation was noted with an asterisk (Figure 4).

For the plasma sample analysis, we performed a non-parametric Mann and Whitney test. The number of TH-positive cells was analyzed with a two-way ANOVA and the *p* value was calculated. Because of the multiplicity of the implemented tests, a Bonferroni *post hoc* correction was applied only followed this test. Statistical analysis was performed using Prism 6.0 software (GraphPad Software Inc., La Jolla, CA, USA).

An unsupervised PCA was first performed on 130 parameters (TLDA, behavior, and plasma data) at different time point for each brain biopsy punches (VTA, NAc, and hypothalamus) to visualize the general structure of the data set (i.e., three global PCA per time point). PCA can be defined as the orthogonal projection of the data onto a lower dimensional linear space, such that the variance of the projected data is maximized in the subspace. We first filtered out genes that are not expressed or slightly expressed (Figure 5). Values for offspring from CD fed dams and from WD fed dams appeared in different colors in individual PCA plots to visualize if these two experimental groups are well separated by the unsupervised PCA components. This analysis segregates the groups of genes that are differentially expressed between the two groups of offspring. Subsequently, focused PCAs were done on different cluster of mRNA markers: plasticity (cell adhesion, cytoskeleton, neurotrophic factor, synaptogenesis, and transcription regulatory), DA pathway, GABAergic pathway, epigenetic modulators (histone deacetylase and histone acetyl transferase). These focused PCAs allow one to visualize simultaneously the correlation between maternal diets and some markers and correlations among specific family genes. A qualitative scale was used for the analysis of the PCA and focused PCA: +++: very good separation; ++: good separation with one rat on the wrong side of the PCA separation; +: quite good separation with two rats (one of each group) on the wrong side, −: no clear separation.

## Results

### Body Weight and Growth

**Figure 2:**
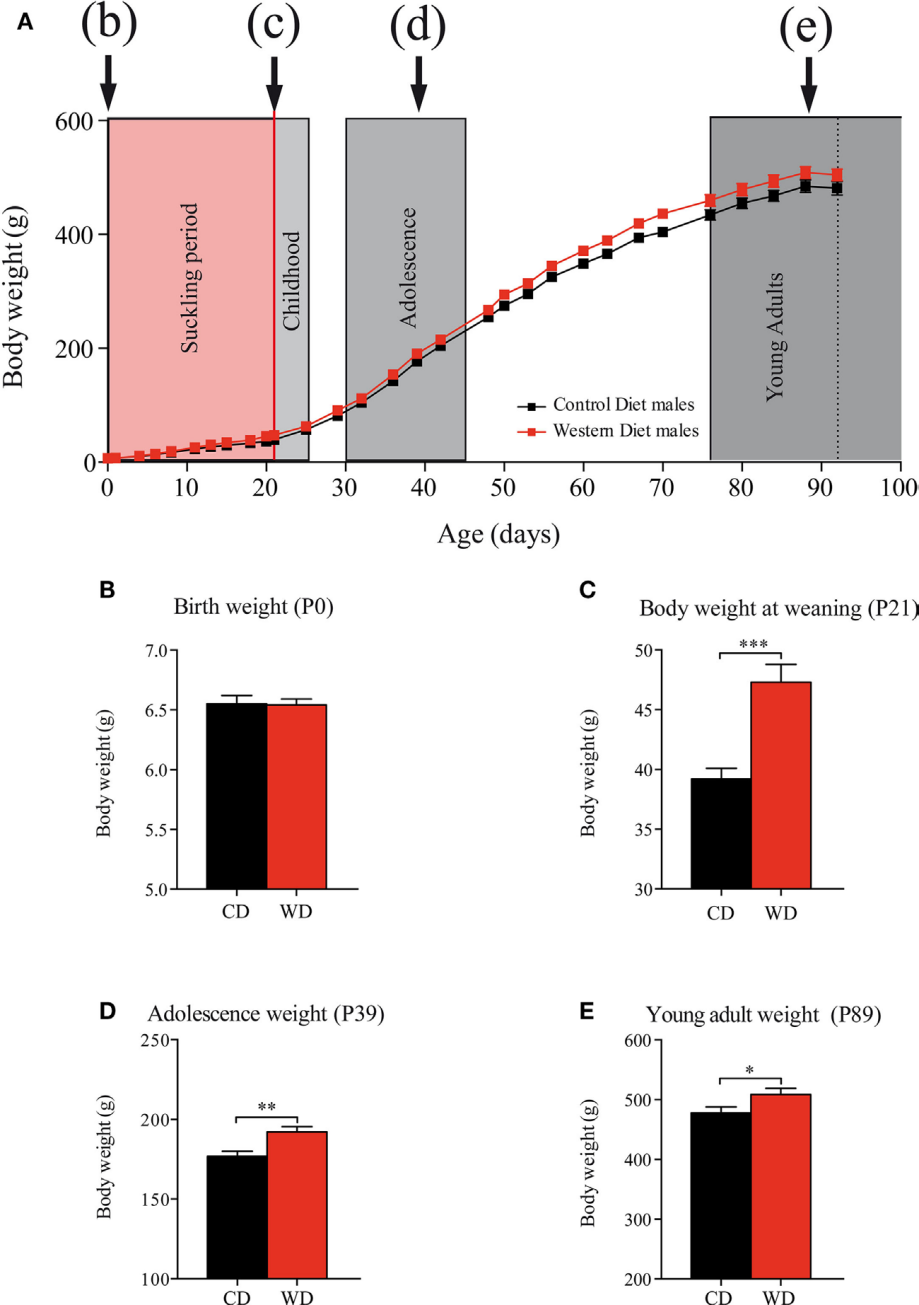
Evolution of offspring body weights from birth to adulthood. **(A)** Body weight day 0 to day 100. Suckling period in red and postweaning periods (c) childhood, (d) adolescence, and (e) young adults in gray. On growth curve, male offspring from control diet (CD) fed dams are represented by black squares and male offspring from western diet (WD) fed dams by red squares. **(B)** Birth weight (Mann and Whitney test *p* = 0.9232). **(C)** Body weight at weaning (MW test, ***p < 0.001). **(D)** Adolescence body weight (MW test, **p = 0.0016). **(E)** Young adult body weight (MW test, *p = 0.0452). Data are expressed as mean ± SEM.

Maternal WD intake during gestation (from G1 to G21) did not affect pups body weight at birth (Figure 2) (CD: 6.55 ± 0.07 g vs WD: 6.54 ± 0.05 g *p* = 0.9232) (Figures 2A,B). Body weight gain from birth to weaning was 21% higher in offspring born from WD dams than offspring from CD dams with a body weight significantly higher at weaning in offspring born from WD dams (36.19 ± 0.90 g vs 47.32 ± 1.48 g *p* < 0.001) (Figure 2C). From weaning to the end of the experiment (P95), the rats were fed with standard chow diet and body weight remained higher for the offspring from WD dams than from CD dams offspring. In details: during adolescence (P39) (Figures 2A,D), CD: 176.8 ± 3.3 g vs WD: 192.2 ± 3.3 g *p* = 0.0016 and at P93 (young adult) (Figures 2A,E) CD: 478 ± 9.9 g vs WD: 508.6 ± 10.3 g *p* = 0.0452.

### Hormones and Metabolic Markers at Different Time Period

Plasma leptin, insulin, glucose, and NEFA concentrations were measured at P25, P45, and P95. At all ages, plasma glucose, NEFA and leptin levels of WD offspring were not statistically different from CD offspring (Table 2, *n* = 6 per group). We observed a significant increase in fat deposition (retroperitoneal fat mass ratio) on offspring from WD fed dams at P25 only (*p* = 0.0327, Mann and Whitney test).

**Table 2:** Retroperitoneal fat mass ratio and plasma dosage: glucose; insulin, NEFA, and leptin.

		Control diet (*n* = 6)	Western diet (*n* = 6)
Glucose (g/l)	P25	1.320 ± 0.036	1.427 ± 0.083
	P45	1.474 ± 0.030	1.461 ± 0.033
	P95	1.349 ± 0.037	1.344 ± 0.042
Insulin (ng/ml)	P25	0.853 ± 0.157	0.831 ± 0.190
	P45	3.585 ± 0.598	1.885 ± 0.484
	P95	4.602 ± 1.544	3.630 ± 0.555
NEFA (g/l)	P25	0.993 ± 0.082	1.460 ± 0.276
	P45	0.669 ± 0.033	0.627 ± 0.041
	P95	1.024 ± 0.101	1.023 ± 0.115
Leptin (ng/ml)	P25	1.593 ± 0.150	1.822 ± 0.499
	P45	4.227 ± 0.565	5.536 ± 0.611
	P95	8.888 ± 0.824	7.956 ± 0.852
Retroperitoneal fat ratio	P25	0.002 ± 0.000	0.004* ± 0.000
	P45	0.009 ± 0.000	0.012 ± 0.002
	P95	0.018 ± 0.001	0.0021 ± 0.003

*The results are expressed as mean ± SEM*.

*Significant difference at p < 0.05.

### Impact of Perinatal WD on Fat Preference from Weaning to Adulthood

**Figure 3:**
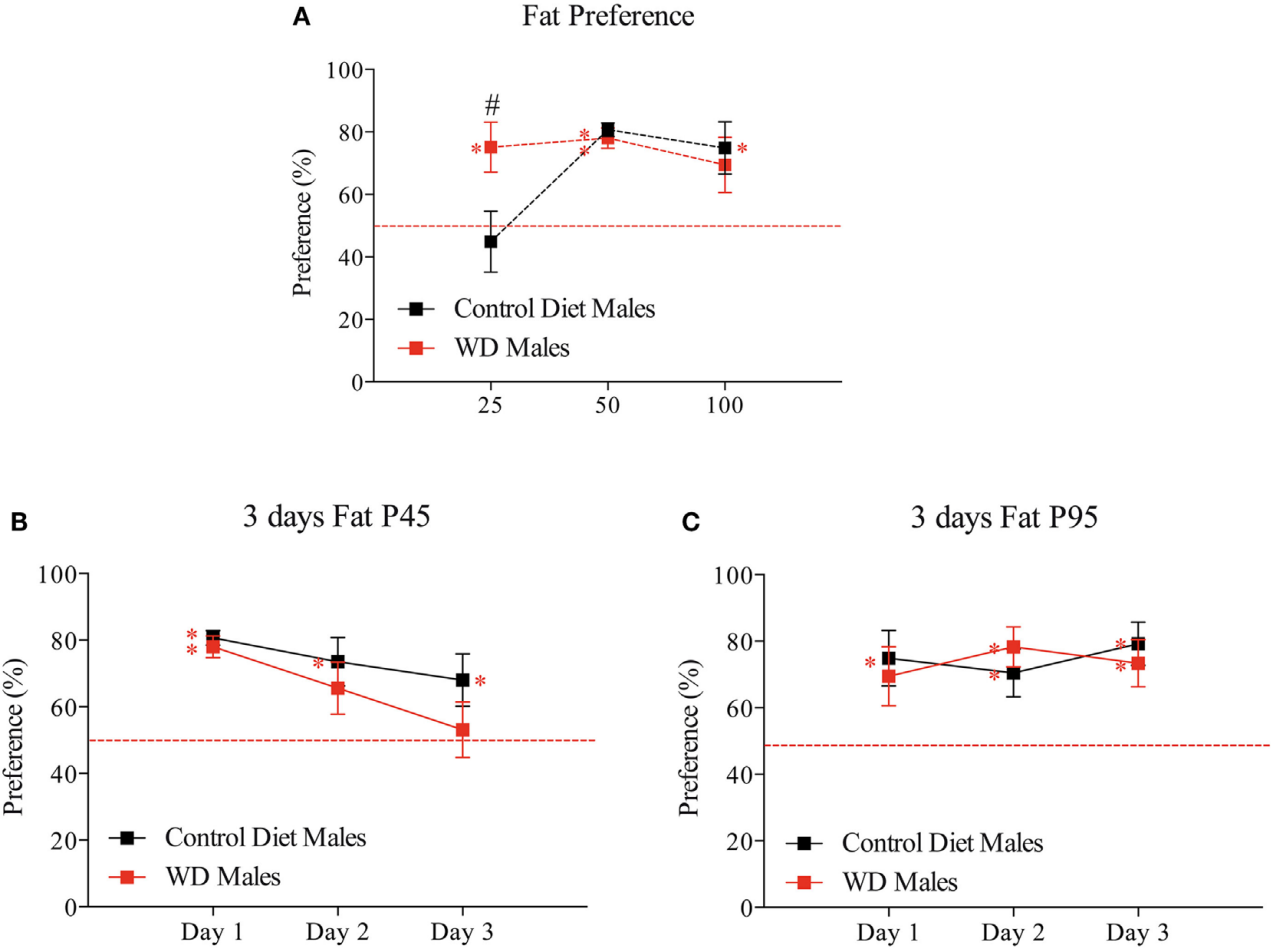
Developmental evolution of fat preference from weaning to adulthood. **(A)** First day fat preference at P25, P45, and P95. Different sets of animals were used at each time point (*n* = 6/group/time point). **(B)** Three consecutive days of fat preference on the same set of animals at P45. **(C)** Three consecutive days of fat preference on the same set of animals at P95. Male offspring from control diet fed dams are represented by black squares and male offspring from western diet (WD) fed dams by red squares. Data are expressed as mean ± SEM, red asterisk *p* < 0.05 Wilcoxon signed-rank test to test if one group values differs from a hypothetical value of 50% corresponding to the absence of preference and black ^#^*p* < 0.05 Mann and Whitney test to test if two group values differ at a time period.

To explore the impact of WD on fat preference, we used a two-bottle choice paradigm at three different time points during growth. This test was used to study specifically the preference for fat taste by avoiding as much as possible the metabolic effect of its ingestion We showed that differences in “extra” calorie intake from the bottle (at P25, P45, and P95) are not statistically significant between groups (Figures S1A–C in Supplementary Material). Moreover the difference in consumption of 1% corn oil solution results in an increase of calorie by 1% for WD rats at P25 (WD: 4.9% vs CD: 3.9% of calories ingested) and 0.5% for CD rats at P45 (WD: 2% vs CD: 2.5% of calories ingested) (Figures S1D–F in Supplementary Material). At P25, pups from CD dams have no preference for fat (44.87 ± 9.8%, *p* = 0.339); on the opposite WD rats present a preference for fat (75.12 ± 8.04%, *p* = 0.039 following Wilcoxon signed-rank test, red asterisk). Moreover there is a statistical difference between the two groups with *p* = 0.0347 (Mann and Whitney test, black hash tag) (Figure 3A).

At P45 and P95, the two groups have a significant preference for fat, i.e., significantly different from the theoretical value of 50% (at P45, CD: 80.68 ± 2.2% *p* = 0.0005 and WD: 78.07 ± 3.25% *p* = 0.0005; at P95, CD: 74.84 ± 8.4% *p* = 0.0425 and WD: 69.42 ± 8.9% *p* = 0.109 following Wilcoxon signed-rank test, red asterisk) (Figure 3A). The values for the two groups were undistinguishable following one day of taste presentation (at P45 *p* = 0.7857 and at P95 *p* = 0.9171 Mann–Whitney test) (Figure 3A).

To know how the rats regulate their fat consumption over time, we repeated the fat presentation for three consecutive days at P45 and P95 (Figures 3B,C). Interestingly at P45, only males from WD dams progressively lost the preference for fat solution (Figure 3B) (third day: 53.12 ± 8.36% *p* = 0.851 following Wilcoxon signed-rank test). However, at P95 (adult age) all animals preferred the fat with no evolution during the 3 days test (Figure 3C).

In summary, in this model, we observed, at early stage (childhood), a preference for fat in rat fed by WD dams with a progressive disinterest over time during adolescence. We observed no difference between the two groups of rats in adulthood.

### Molecular Signature of Brain Plasticity and GABA Circuits Remodeling in the Hypothalamus and Reward Pathways

To determine whether maternal WD intake during gestation and lactation has an impact on the hypothalamus and reward pathways of the offspring, we measured the relative expression of several key factors of brain plasticity, brain modeling, and markers of neuronal circuits implicated in food intake and epigenetic regulators. We used TLDA to analyze their abundance in different brain area (i.e., hypothalamus, VTA, and NAc) (Table S1 in Supplementary Material) at the three time periods. Screening was performed after the two-bottle choice tests at P25, P45, and P95 (Figure 1) on six males born from WD fed dams and six males born from CD fed dams.

**Figure 4:**
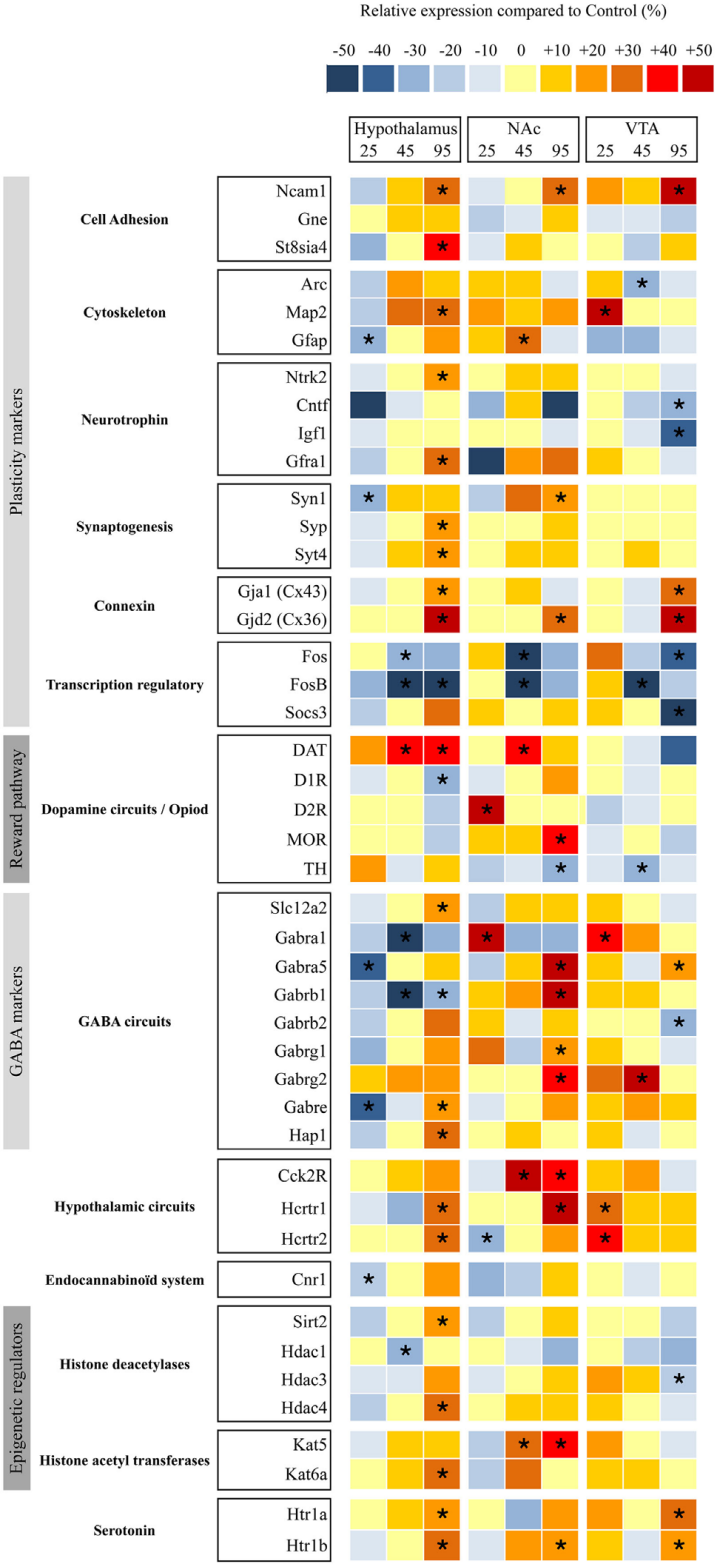
Relative gene expression in nucleus accumbens (NAc), ventral tegmental area (VTA), and hypothalami from perinatal-western diet fed rats and perinatal-control diet fed rats at three time periods. Simultaneous quantification of the expression of genes in hypothalami, NAc, and VTA biopsies using TaqMan low-density arrays. Individual analysis was conducted for each gene. Significant difference at *< 0.05.

At P25 in hypothalamus, five genes from thirteen different categories displayed a significantly lower mRNA expression level mainly in plasticity markers and GABA markers ranging between −20% (Gfap) and −40% (Gabra5) in pups from WD fed dams compared with rats from CD fed dams. In reward pathway biopsies (VTA and NAc), two genes displayed a statistical higher mRNA expression levels (D2R and Gabra1), i.e., DA signaling and GABA receptors and one gene a lower expression (Hcrtr2) (i.e., orexin 2 receptor) in NAc, whereas four genes displayed a significantly higher mRNA expression level (Map2, Gabara1, Hcrtr1, and Hcrtr2) (i.e., plasticity markers, GABA receptors and serotoninergic receptors) in VTA (Figure 4).

At P45 in hypothalamus, five genes from thirteen different categories displayed a lower mRNA expression level ranging between −20% (Fos) and −50% (FosB) in pups from WD fed dams compared with rats from CD fed dams. At P45 in reward pathway biopsies, four genes displayed a higher mRNA expression level (Gfap, Dat, Cck2r, and Kat5) and two genes a lower expression (Fos and FosB) in NAc whereas three genes displayed a lower mRNA expression level (Arc, FosB, and Th) and one gene a higher level (Gabrg2) in VTA.

At P95 in hypothalamus, 20 genes from thirteen different categories displayed a higher mRNA expression level ranging between +20 and +40% (Syt4 to Gjd2) and 3 genes displayed a lower mRNA expression (FosB, D1r, and Gabarb1) in pups from WD fed dams compared with rats from CD fed dams. At P95 in reward pathway biopsies, 12 genes displayed a higher mRNA expression level ranging between +20 and +40% (Syn1 to Hcrt1) and 1 gene a lower expression (Th) in NAc, 6 genes displayed a higher mRNA expression level (Ncam1, Gja1, Gjd2, Gabra5, Htr1a, and Htr1b), and 6 genes displayed a lower mRNA expression level (Cntf, Igf1, Fos, Socs3, Gabrb2, and Hdac3) in VTA.

We then performed three unsupervised PCA corresponding to the three brain biopsies using all the quantified parameters (i.e., plasma dosage, behavioral data and mRNA expression variations). A clear separation of the two groups was obtained only at P95 for NAc and VTA (Table 3).

**Table 3:** Principal component analysis (PCA) synthesis: qualitative analysis of PCA group separation for global PCA and focused PCA.

	PCA					
	P25		P45		P95	
	Global PCA	Focused PCA	Global PCA	Focused PCA	Global PCA	Focused PCA
Nucleus accumbens	−	Dopamine++	−	−	+++	γ-Aminobutyric acid (GABA)+++, epigenetic+, plasticity+
Ventral tegmental area	−	−	−	−	++	Epigenetic++, plasticity+
Hypothalamus	−	Plasticity++	−	−	−	GABA+++, plasticity+

*A qualitative scale was used for the analysis of the PCA and focused PCA: +++: very good separation; ++: good separation with one rat on the wrong side of the PCA separation; +: quite good separation with two rats (one of each group) on the wrong side, −: no clear separation*.

**Figure 5:**
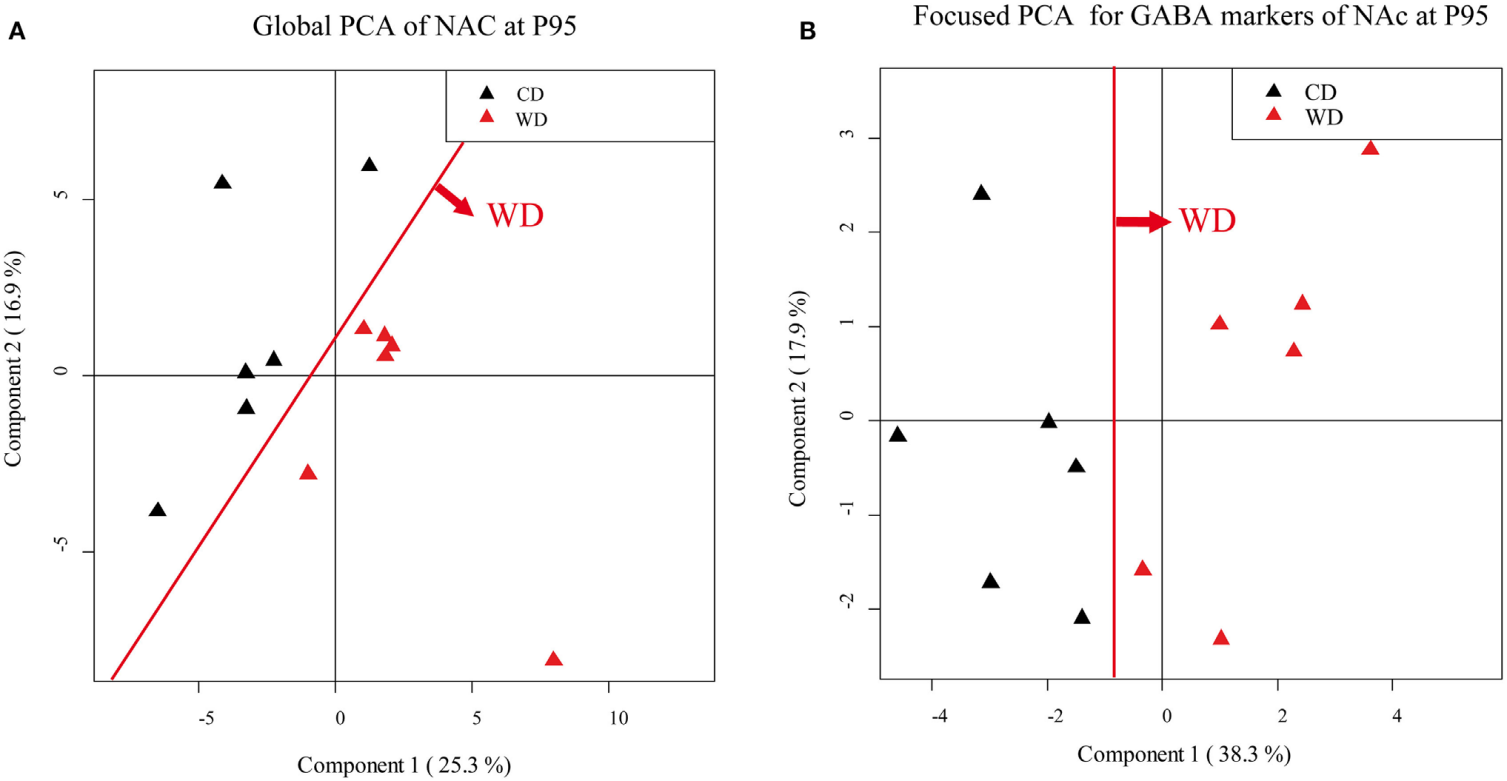
Principal component analysis (PCA). Score scatter plot of PCA **(A,B)**. **(A)** Global PCA from nucleus accumbens (NAc) samples of P95 rat males. Black triangles correspond to offspring from control diet (CD) fed dams and red triangles correspond to offspring from western diet (WD) fed dams. **(B)** Focused PCA for γ-aminobutyric acid (GABA) markers, from NAc samples of P95 rat males. Black triangles correspond to offspring from CD fed dams, and red triangles correspond to offspring from WD fed dams. The red line shows the clear separation between the groups.

According to the PCA correlation circle and the TLDA data (representing the majority of the variables included in this PCA), we defined the gene families that could be responsible for the segregation and performed a focused PCA (Figures 5A,B, for example). The focused PCA revealed that at P25 DA markers in NAc and plasticity markers in hypothalamus could separate the two groups of offspring (Table 3 for summary). No such discrimination was then obtained at P45. However, the same analysis at P95 revealed that the different markers of the GABA system in NAc and hypothalamus, plus the plasticity markers (in hypothalamus, NAc and VTA) and epigenetic regulators (only in NAc) contribute to separate the two groups of animals (Figure 5; Table 3).

This analysis reveals the long-lasting influence of perinatal diet on GABAergic markers as well as plasticity and epigenetic markers in both the homeostatic and the reward pathway implicated in feeding behavior.

### Immunohistochemistry of TH Cells Confirmed Transcript Analysis

**Figure 6:**
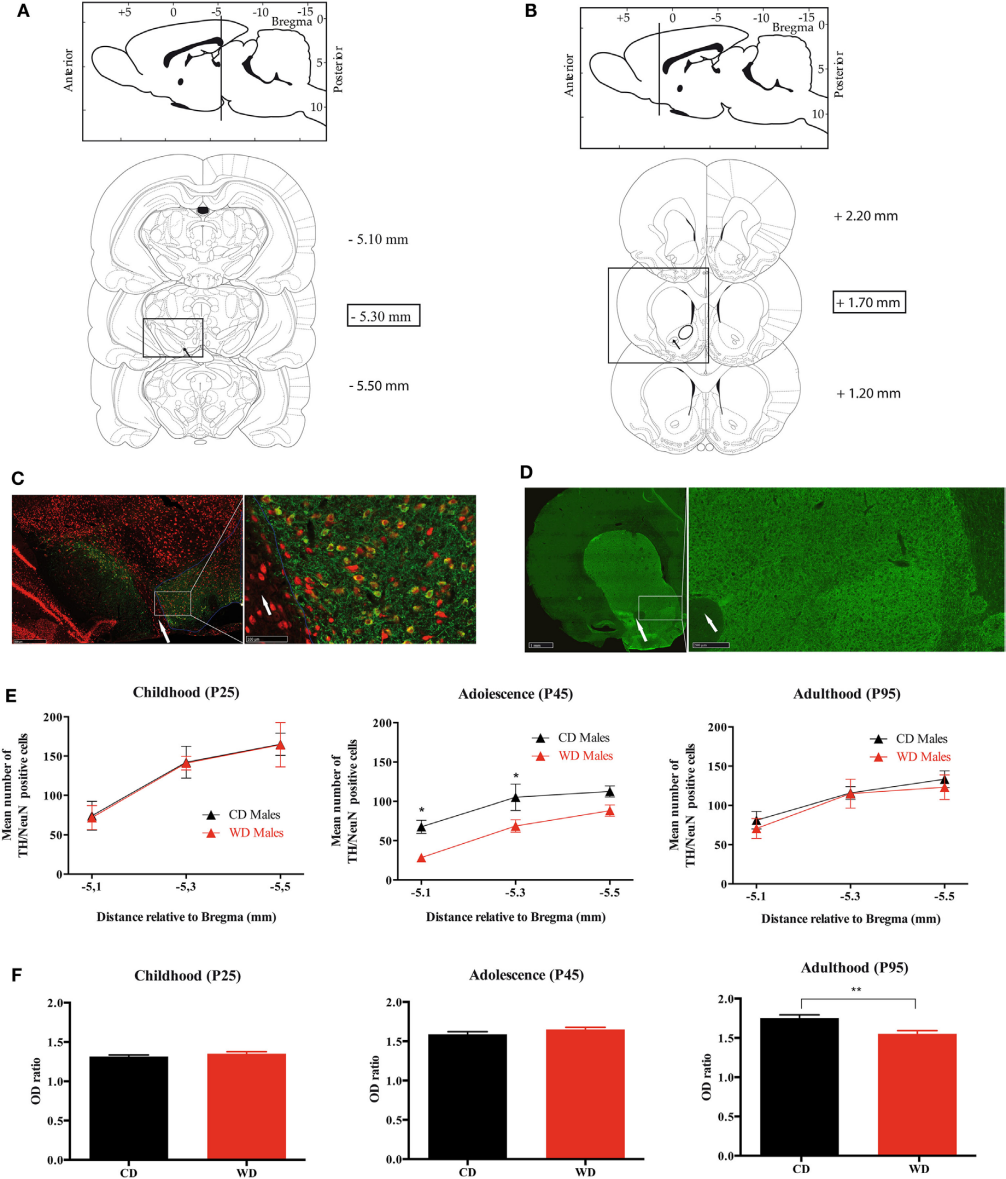
Quantification of TH/NeuN positive neurons in ventral tegmental area (VTA) and TH density fibers in nucleus accumbens (NAc) from weaning to adulthood in offspring from western diet (WD) or control diet (CD) fed dams. **(A)** Scheme from Paxinos and Watson’s atlas. Upper panel: sagittal view of the antero-posterior level of the VTA. Lower panels: the three rostrocaudal levels quantified for TH (+)/NeuN(+) immunostaining. The black arrow shows the exit of the third nerve that is used as anatomical landmark. The scale on the right corresponds to the rostrocaudal section localizations in millimeters from Bregma. **(B)** Scheme from Paxinos and Watson’s atlas. Upper panel: sagittal view of the antero-posterior level of the NAc. Lower panels: the three rostrocaudal levels quantified for TH fibers density immunostaining. The scale on the right corresponds to the rostrocaudal section localizations in millimeters from Bregma. **(C)** Photomicrograph of TH/NeuN immunostaining at the level of the VTA, −5.30 mm from Bregma. Red labeling is for NeuN, and green one for TH. The white arrow shows the exit of the third nerve. The white circle delimitates the VTA localization. **(D)** Photomicrograph of TH immunostaining at the level of the NAc, +1.70 mm from Bregma. Green labeling is for TH. The white arrow shows the anterior commissure. **(E)** Three graphs (childhood, adolescence, and adulthood) of TH/NeuN quantification at three rostrocaudal levels of the VTA. Black triangles indicate the mean counts from the VTA of the CD dams offspring and the red triangles indicate the mean counts from the VTA of the WD dams offspring. *Significant difference at *p* < 0.05. **(F)** Three graphs (childhood, adolescence, and adulthood) of TH fibers density at three rostrocaudal levels of the NAc. Black plots indicate the mean optical density (OD) ratio of the CD dams’ offspring, and the red plots indicate the mean OD ratio of the NAc WD dams’ offspring. **Significant difference at *p* < 0.01.

Because we observed some variation in TH mRNA in NAc and VTA at the various developmental periods, we aimed at correlating these results with TH immunostaining. The number of TH/NeuN positive cells was analyzed in the VTA where dopaminergic cell bodies are located and the OD of TH immunolabeling was quantified in the nerve endings located in the NAc. TH (+) cells were less abundant in the VTA of WD compared to CD rats at P45 only (Figures 6A,C,E; Figure S2A in Supplementary Material). There was no significant interaction between section level and the TH/NeuN quantification at the three periods (P25 *p* = 0.9991, P45 *p* = 0.9026, and P95 *p* = 0.9170). At P45 only, a statistical difference was obtained between the two offspring groups (*p* = 0.0002) (Figure 6E). In addition, we observed no difference in OD of TH immunostaining in the NAc at P25 and P45 between the two groups (OD ratio values at P25: 1.314 ± 0.022 in CD vs 1.351 ± 0.026 in WD, *p* = 0.2681; OD ratio values at P45: 1.589 ± 0.033 in CD vs 1.651 ± 0.027 in WD, *p* = 0.1542). However, a significant decrease of OD of TH nerve endings was found in NAc from WD group at P95 (OD ratio values at p95: 1.752 ± 0.041 in CD vs 1.550 ± 0.046 in WD, *p* = 0.0037) (Figures 6B,D,F; Figure S2B in Supplementary Material).

## Discussion

**Figure 7:**
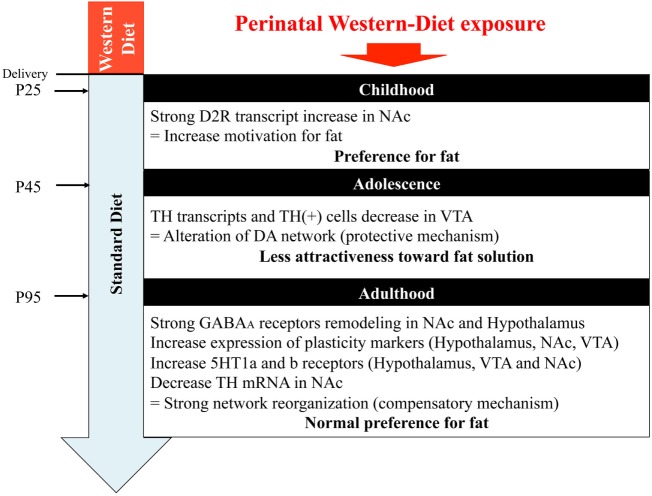
Graphical abstract. NAc, nucleus accumbens; VTA, ventral tegmental area.

In this study, we hypothesized that maternal perinatal overnutrition will influence the program of development of reward pathways involved in energy homeostasis, food choice, and food intake of the offspring. We extensively examined the impact of maternal WD intake from birth to weaning on GABA, serotonin, and DA pathways of specific brain areas (VTA, NAc, and hypothalamus) in the offspring, from childhood to adulthood. Our results suggest that the use of a diet, rich in fat and sweet, strictly restricted to the perinatal period has an impact on early fat preference (childhood) in the offspring correlated with change in gene expression profile and neuroanatomical/architectural changes of the mesolimbic dopaminergic networks. However, when the offspring were kept under chow diet, we observed in adolescent WD fed rats a progressive loss of attractiveness toward fat that was correlated with a reduced expression of genes of the DA system and a slight reduction of TH-positive neurons in the VTA. Later in life fat preference was not different between groups even though an important plasticity of the GABAergic networks and of the energy homeostasis network of the hypothalamus was identified in rat from WD fed dams (Figure 7).

The first impact of perinatal-WD intake that we observed in this study is an increased body weight of the offspring at weaning but no difference at birth. Indeed, animals of the WD group gain 21% more weight than CD at the end of the sucking period. Previous studies have provided conflicting results regarding change in birth weight for offspring from WD fed dams: a higher body weight (19, 34), a lower body weight (18, 21, 35) or no difference (6, 22). Our data are in line with a recent meta-regression analysis (36) conducted on 171 experimental publications that concluded that maternal HFD exposure did not affect offspring birth weight but induced an increased body weight at the end of the lactation period. The higher body weight of the WD offspring probably reflects a change in milk composition and/or milk production that has been illustrated in previous publications (37, 38). In accordance with their higher body weight, the retroperitoneal fat ratio of the WD offspring was significantly higher than that of the CD offspring at the end of the suckling period (P25, Table 2), which is also consistent with previous studies (18, 21). However, the higher adiposity did not persist at P45 and P95, and other metabolic parameters as insulin, NEFA, and glucose plasma were not different between groups. Our results demonstrated that without a clear maternal obesity during gestation and lactation, the diet by itself is not sufficient to induce lasting metabolic effects in the offspring (22, 39, 40).

It has been reported that perinatal HFD intake positively correlate with offspring preference for palatable food (41). In our study, we performed a longitudinal study aiming at testing fat preference on offspring that are weaned on regular chow.

### Impact of Perinatal WD on Childhood (after Weaning)

Rodents pups eat solid food 19–20 days after birth (42) when their cerebral reward pathways are not yet mature (17). It was therefore very interesting to study their very early preference for fat and correlate this early preference with brain transcripts analysis. Just after weaning, we observed a preference for fat in WD offspring that was not evidenced in CD rats. This is in line with others reports showing a link between perinatal malnutrition and palatable food preference and a low preference for fat at early age for control rats (43).

The global PCA did not allow discriminating the group of pups in respect to maternal diet at that age. However, when a targeted PCA, restricted to DA markers, was performed, we obtained a good segregation of the groups. Indeed, there is a marked increase in the expression of the D2 receptor mRNAs in the NAc in WD pups. This postsynaptic D2 overexpression in the NAc could be partly involved in a higher motivation for fat (44). Few other transcripts are modified in WD pups compared to CD pups, such as an increase of alpha 1 GABAA subunit in NAc and VTA and a decrease of alpha 5 GABAA subunit in the hypothalamus that suggests a reorganization of GABAA receptors in these nuclei.

### Impact of Perinatal WD on Adolescence

At P45, we observed a similar high-fat preference for both groups on the first day of presentation but, interestingly, the WD rats progressively lost their interest for fat after repeated presentation. The adolescence is a critical period of neurobehavioral reorganization necessary for life-long cognitive processing (45), and various studies showed a marked vulnerability to detrimental cognitive effect of a fat diet (46–48). This result is in apparent contradiction with previous work of the group of Muhlhausler (21, 35) in which juvenile rats (6 weeks) showed a clear preference for junk food. However, in their publications the experimental paradigm was different since the rats had free access to both standard chow and junk food from weaning to the sacrifice (6 weeks).

Concomitantly, we measured an increase of Dat mRNA in the NAc and a decrease of Th mRNA in the VTA that was confirmed by the immunohistochemistry that showed a reduced TH (+) cells number in the VTA of WD rats. After an elevated transcriptomic activity for the DA system at weaning, the reduced activity at P45 may explain the low interest for the palatable food observed in our WD rats. It should also be noted that the systematic decrease of Fos and FosB mRNA expression in the various nuclei that we analyzed could be a mark of a reduced cerebral activity after maternal WD exposure.

Adolescent WD rats showed a more rapid disinterest for fat that is opposite to their earlier behavior. The use of a “normal” diet during childhood seems to “protect” them toward an exaggerated fat preference at adolescence. On the contrary when rats have free access to junk food after weaning, as in Ref. (21, 35), they demonstrate at adolescence a strong preference for fat. This result suggest that 3 weeks chow diet after weaning could have reprogrammed the circuits and make the adolescent offspring less sensitive to an acute fat challenge.

### Impact of Perinatal WD on Adults

Adult rats no longer displayed difference of preference for fat, even after repeated fat presentation as already described (22, 35). Concomitantly, we observed a decrease in Th mRNA and protein in the NAc, and a tendency for a reduced expression of Dat mRNA in the VTA. Naef and coworker (20) already reported a low activity of the DA system in adult rats fed in perinatal period with an HFD, with a blunted DA response to amphetamine measured with microdialysis and an increase motivation for fat reward (see table that summarized recent qPCR data on this model, Table S2 in Supplementary Material). One limitation of TH quantification (mRNA and immunohistochemistry) in NAc comes from the fact that NAc cells could also express Th mRNA and protein and then could bias the DA fibers quantification (49, 50). However, the use of TH immunostaining in NAc mainly revealed the dense axon terminals coming from midbrain DA neurons (VTA and SNc). Usually, the TH expressing neurons in the striatum and NAc could be discerned only in highly DA lesioned animals (51) and therefore could be hardly detectable in our immuno-sections. In this study, we also observed a strong increase in mu opioid receptor in NAc when other groups, with different models, showed a decrease expression in the ventral striatum of rat early exposed to HFD (during lactation and gestation) (19, 21) or no change (35). These modifications, measured at the mRNA level only, could reflect a slight hypo activity of the DA circuits associated with a higher opioid sensitivity (52) which probably are not sufficient to have an impact on the behavioral test we carried out. These assumptions need to be confirmed using functional approaches. In a recent paper, with a similar model, Romani-Perez et al., were not able to observe a significant increase of motivation in operant conditioning boxes for HFD offspring but observed a shorter latency to reach a goal box in a runway test paradigm (22). Despite the absence of long-lasting fat preference in our experimental conditions, we found that perinatal maternal WD intake has a long-lasting effect on other cerebral circuits mostly mediated by GABA remodeling in NAc and Hypothalamus. NAc is considered as a “sensory sentinel” for consummatory behavior (52). Recent studies have shown that food intake was suppressed by inhibition of GABA-releasing LH neurons (53). O’Connor et al. showed that NAc D1R neurons (GABAergic projecting neurons) selectively inhibit LH VGAT neurons to stop food intake (54). These experiments unveil a GABA circuit (NAc/Hypothalamus) that may be responsible for controlling behavioral response. This ventral striatum–hypothalamic system complements another circuit that involves the bed nucleus stria terminalis GABA-releasing VGAT projecting neuron to the glutamate releasing Vglut LH neurons and direct inhibition of LH vglut2 elicits feeding (55). Another important component of the appetite-regulating circuit that involves NAc shell is a GABA-releasing inhibitory projection to the VP (56). These data highlight the crucial role of GABA signaling in the interplay between hypothalamus and NAc to promote feeding. In our study, we were not able to discriminate the population of neurons involved in the GABA remodeling and how these modifications could alter the networks. However, the central role of GABA circuits deserves more interest. In particular, it would be very interesting to perform further functional experiments of these GABA circuits using electrophysiological approaches (57). We also observed a global upregulation of mRNA transcript for 5HT1a and 5HT1b receptors in the three nuclei studied. The majority of projecting serotonin fibers comes from the dorsal raphe nucleus (DRN) and median raphe nucleus (MRN). Recent data from *in vivo* recordings and imaging studies showed a positive role of 5HT in reward (58). 5HT fibers from DRN are involved in impulsivity control (59). Increase 5HT1a in VTA and NAc could be a compensatory mechanism that could control impulsivity. In hypothalamus, pharmacological studies suggest that 5HT1a receptor subtypes may suppress feeding behavior induced by serotonin stimulation (60, 61). Increased 5HT1a and b receptors in hypothalamus could potentiate the feeding-suppressive action of serotonin and therefore could constitute a compensatory mechanism. These assumptions need to be verified by performing proper functional experiments.

These networks changes are associated with modifications of plasticity markers as Ncam mRNA. In the hypothalamus of adult rats, we observed an increased in Ncam1 and St8sia4 transcripts suggesting and increase in polysialic acid (PSA) signaling. PSA is a cell-surface glycan that modulates cell-to-cell interactions. Polysialylation of cell adhesion proteins is involved in various synaptic plasticity-dependent processes in the central nervous system and has been reported to be required for the adaptive synaptic plasticity of feeding circuits during acute positive energy balance (33, 62). In addition other regulators of cell interaction and synaptogenesis might be involved in this hypothalamic plasticity.

In conclusion (Figure 7), maternal WD intake has a long-lasting influence on the organization of the homeostatic and hedonic circuits regulating eating behavior in the offspring. By the analysis of three critical time periods, we were able to show a clear evolution for fat preference correlated with specific brain molecular signatures. During childhood, the preference for fat might be correlated with a higher activity of the DA system. Adolescence, characterized by an inversion of fat preference, was associated with lower expression of DA system markers suggesting a compensatory mechanism. A very interesting point to notify is that, in this model, a balanced diet after weaning could protect adolescent rat from deleterious feeding habits by reducing their desire for fat. Although in adulthood the two groups have a similar high preference for fat, rats from WD fed dams showed a profound remodeling of the GABA circuits. What are the consequences of this lasting plasticity? Will an exaggerated obesogenic diet intake during adolescence reactivate this blunted reward system? Such questions could be relevant in the nutritional follow-up of new born and children risen in westernized countries.

## Ethics Statement

All experiments were performed in accordance with the guidelines of the local animal welfare committee, the EU (directive 2010/63/EU), the Institut National de la Recherche Agronomique (Paris, France), and the French Veterinary Department (A44276). The experimental protocol was approved by the institutional ethic committee and registered under reference APAFIS 8666. Every precaution was taken to minimize stress and the number of animals used in each series of experiments.

## Author Contributions

JP and PB performed experiment and participate in the discussion and writing. TM performed the PCA and participated in discussion and writing. SN contributed to the design of the experiment and participated in discussion. PP contributed to the design of the experiment, participated to the discussions, and wrote the manuscript. VP designed and performed the experiments, analyzed the data, and wrote the manuscript.

## Conflict of Interest Statement

The authors declare that the research was conducted in the absence of any commercial or financial relationships that could be construed as a potential conflict of interest.

## Supplementary Material

Figure S1 Total energy intake from corn oil containing bottle. **(A)** Calories intake from the corn oil bottle for 24 h at P25 in pups from western diet (WD) fed dams and pups from control diet (CD) fed dams. **(B)** Calories intake from the corn oil bottle for 24 h at P45 (the third day of bottle test). **(C)** Calories intake from the corn oil bottle for 24 h at P95 (the third day of bottle test). For panels **(A–C)**, data are expressed as mean ± SEM, no statistical difference (*p* > 0.05) was observed, following Mann and Whitney non-parametric test, at all ages. **(D)** Percentage of calories intake from the corn oil bottle compare to the total calories intake (corn oil bottle + standard chow diet) for 24 h at P25 in WD pups and CD pups. **(E)** Percentage of calories intake from the corn oil bottle compare to the total calories intake (corn oil bottle + standard chow diet) for 24 h at P45 (the third day of bottle test) in WD pups and CD pups. **(F)** Percentage of calories intake from the corn oil bottle compares to the total calories intake (corn oil bottle + standard chow diet) for 24 h at P95 (the third day of bottle test) in WD pups and CD pups. For panels **(D,E)**, data are expressed in percent of total calorie intake no statistical difference (*p* > 0.05) was observed, following chi-square with Yates’ correction, at all ages.

Figure S2 Representative photomicrographs of TH immunostaining in nucleus accumbens (NAc) and ventral tegmental area (VTA) at three different time points. **(A)** Photomicrograph of TH/NeuN immunostaining at the level of the VTA, −5.30 mm from Bregma. Red labeling is for NeuN, and green one for TH. The white arrow shows the exit of the third nerve. **(B)** Photomicrograph of TH immunostaining at the level of the NAc, +1.70 mm from Bregma. Green labeling is for TH. The white arrow shows the anterior commissure.

Table S1 TaqMan low-density array gene list with the corresponding life technologies inventoried codes.

Table S2 Summary of published data concerning dopamine pathway transcripts expression. The red characters correspond to childhood period, the blue ones to adolescence, and the black ones to adult. =: corresponds to a similar transcript expression between groups, +: corresponds to a higher transcript expression in pups from high caloric diet [junk food, western diet (WD), or high-fat diet (HFD)] fed dams, and −: corresponds to a lower transcript expression in pups from high caloric diet (junk food, WD or HFD) fed dams.
