# Supplementary figures and images for: Perinatal Western Diet Consumption Leads to Profound Plasticity and GABAergic Phenotype Changes within Hypothalamus and Reward Pathway from Birth to Sexual Maturity in Rat
Source: Front Endocrinol (Lausanne). 2017 Aug 29;8:216. doi: 10.3389/fendo.2017.00216 (PMC5581815; doi:10.3389/fendo.2017.00216)

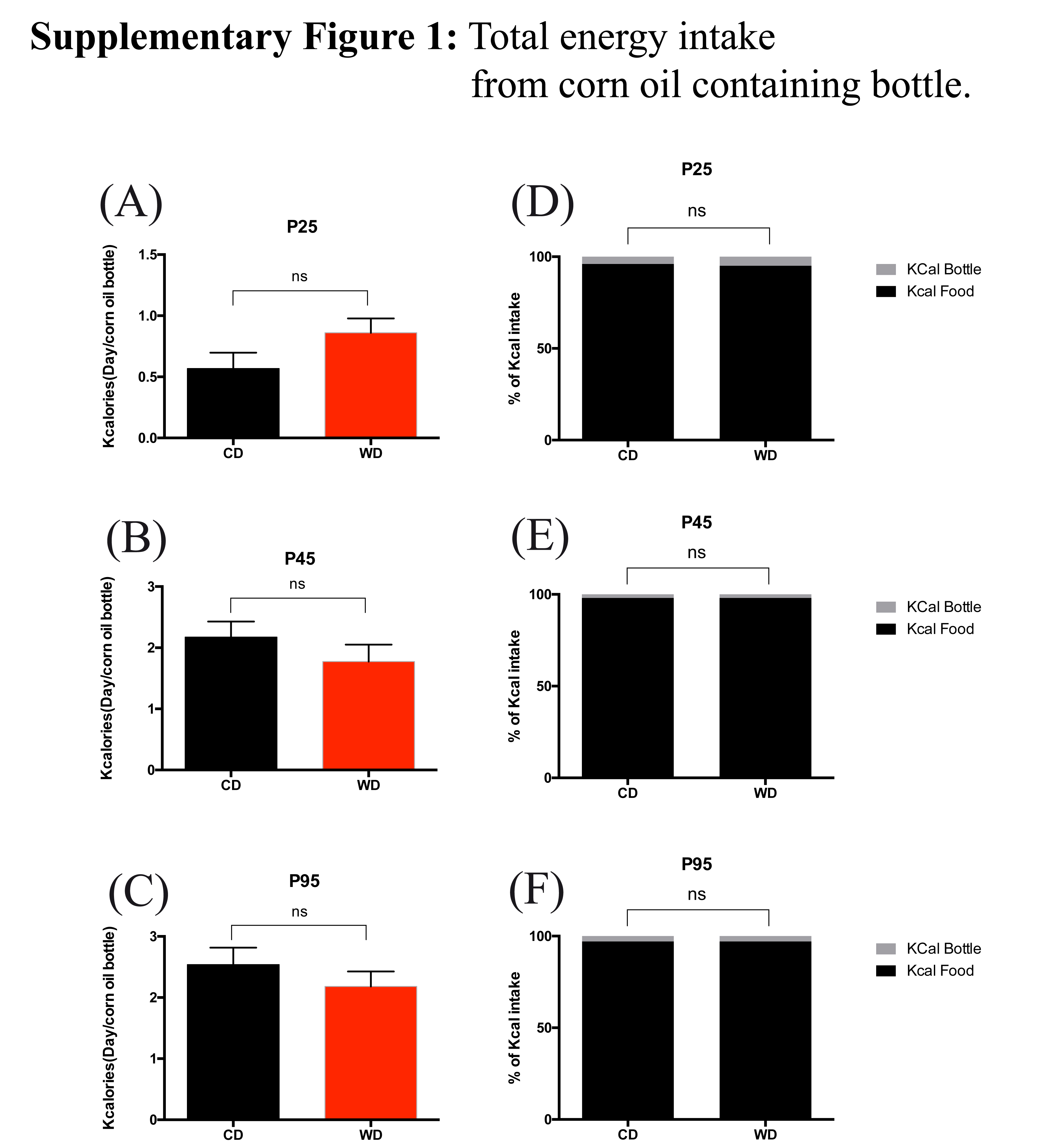

Supplement: Figure S1 — Total energy intake from corn oil containing bottle. (A) Calories intake from the corn oil bottle for 24 h at P25 in pups from western diet (WD) fed dams and pups from control diet (CD) fed dams. (B) Calories intake from the corn oil bottle for 24 h at P45 (the third day of bottle test). (C) Calories intake from the corn oil bottle for 24 h at P95 (the third day of bottle test). For panels (A–C), data are expressed as mean ± SEM, no statistical difference (p > 0.05) was observed, following Mann and Whitney non-parametric test, at all ages. (D) Percentage of calories intake from the corn oil bottle compare to the total calories intake (corn oil bottle + standard chow diet) for 24 h at P25 in WD pups and CD pups. (E) Percentage of calories intake from the corn oil bottle compare to the total calories intake (corn oil bottle + standard chow diet) for 24 h at P45 (the third day of bottle test) in WD pups and CD pups. (F) Percentage of calories intake from the corn oil bottle compares to the total calories intake (corn oil bottle + standard chow diet) for 24 h at P95 (the third day of bottle test) in WD pups and CD pups. For panels (D,E), data are expressed in percent of total calorie intake no statistical difference (p > 0.05) was observed, following chi-square with Yates’ correction, at all ages. [file image_1.jpeg]

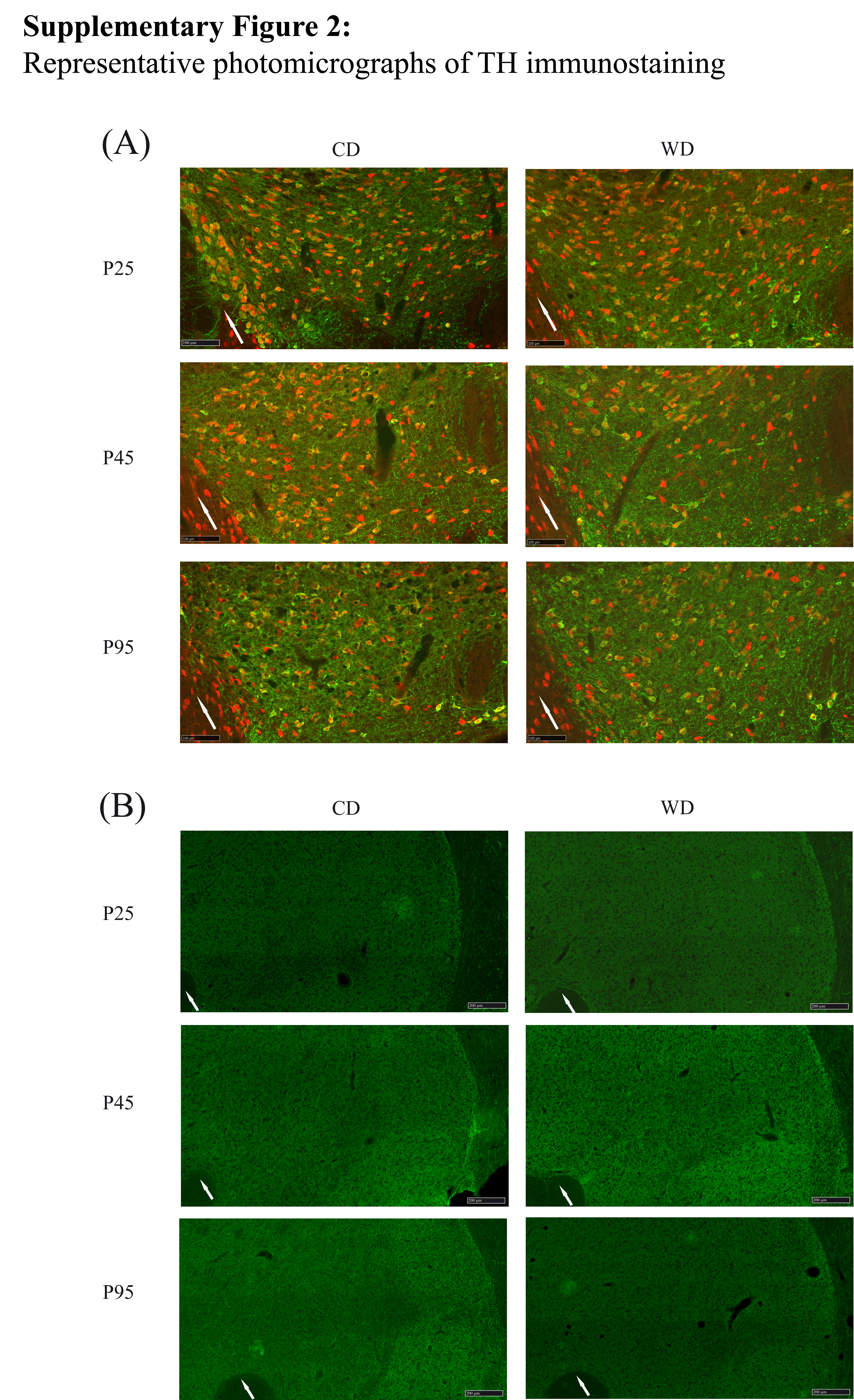

Supplement: Figure S2 — Representative photomicrographs of TH immunostaining in nucleus accumbens (NAc) and ventral tegmental area (VTA) at three different time points. (A) Photomicrograph of TH/NeuN immunostaining at the level of the VTA, −5.30 mm from Bregma. Red labeling is for NeuN, and green one for TH. The white arrow shows the exit of the third nerve. (B) Photomicrograph of TH immunostaining at the level of the NAc, +1.70 mm from Bregma. Green labeling is for TH. The white arrow shows the anterior commissure. [file image_2.jpeg]
