# Supplementary material for: Perinatal Western Diet Consumption Leads to Profound Plasticity and GABAergic Phenotype Changes within Hypothalamus and Reward Pathway from Birth to Sexual Maturity in Rat
Source: Front Endocrinol (Lausanne). 2017 Aug 29;8:216. doi: 10.3389/fendo.2017.00216 (PMC5581815; doi:10.3389/fendo.2017.00216)
Supplement: Table S1 — TaqMan low-density array gene list with the corresponding life technologies inventoried codes. [file table_1.pdf]

Supplementary Table 1: *TaqMan Low Density Array (TLDA) gene list*

Gene number	Type	Gene Symbol	Reference Life Technologies
1	Cell Adhesion	**Ncam1**	Rn00580526_m1
2		**St8sia4**	Rn01427310_m1
3		**Gne**	Rn00587161_m1
4	Cytoskeleton	**Arc**	Rn00571208_g1
5		**Map2**	Rn00565046_m1
6		**Gap43**	Rn01474579_m1
7		**Gfap**	Rn00566603_m1
8	Neurogenesis	**Nes**	Rn00564394_m1
9		**Pcna**	Rn01514538_g1
10		**Mki67**	Rn01451446_m1
11	Neurotrophin	**Bdnf**	Rn02531967_s1
12		**Ntrk2**	Rn01441749_m1
13		**Cntf**	Rn00755092_m1
14		**Ntf3**	Rn00579280_m1
15		**Ntrk3**	Rn00570389_m1
16		**ret**	Rn01463098_m1
17		**Gfra1**	Rn01444617_m1
18		**Gdnf**	Rn00569510_m1
19		**Igf1**	Rn00710306_m1
20	Synaptogenesis	**Snap25**	Rn00578534_m1
21		**Syn1**	Rn00569468_m1
22		**Syp**	Rn00561986_m1
23		**Syt4**	Rn01157571_m1
24	Connexin	**Gja1**	Rn01433957_m1
25		**Gjd2**	Rn00439121_m1
26	Transcription regulatory	**Fos**	Rn00487426_g1
27		**FosB**	Rn00500401_m1
28		**Socs3**	Rn00585674_s1
29	Dopamine circuits / opioid	**Comt**	Rn01404927_g1
30		**Slc6a3**	Rn00562224_m1
31		**Th**	Rn00562500_m1
32		**Ppp1r1b**	Rn01452984_m1
33		**DRD1**	Rh02902958_m1
34		**Drd1a**	Rn03062203_s1
35		**Drd2**	Rn00561126_m1
36		**Drd3**	Rn00567568_m1
37		**Drd4**	Rn00564071_m1
38		**Drd5**	Rn00562768_s1
39		**Oprm1**	Rn01430371_m1
40		**Penk**	Rn00567566_m1
41	GABA circuit	**Slc12a5**	Rn00592624_m1
42		**Slc12a2**	Rn00582505_m1
43		**Gabra1**	Rn00788315_m1
44		**Gabra2**	Rn01413643_m1
45		**Gabra3**	Rn01294271_m1
46		**Gabra4**	Rn00589846_m1
47		**Gabra5**	Rn00568803_m1
48		**Gabra6**	Rn00573029_m1
49		**Gabrb1**	Rn00564146_m1
50		**Gabrb2**	Rn00564149_m1
51		**Gabrb3**	Rn00567029_m1
52		**Gabrd**	Rn01517017_g1
53		**Gabrg1**	Rn00589841_m1
54		**Gabrg2**	Rn00788325_m1
55		**Gabrg3**	Rn00577639_m1
56		**Gabre**	Rn00576522_m1
57		**Hap1**	Rn00577100_m1

Gene number	Type	Gene Symbol	Reference Life Technologies
58	Hypothalamic circuit	**Hcrtr1**	Rn00565032_m1
59		**Hcrtr2**	Rn00565155_m1
60		**Ghrl**	Rn00572319_m1
61		**Ghsr**	Rn00821417_m1
62		**Cckbr**	Rn00565867_m1
63	Homeostasis regulation	**InsR**	Rn00690703_m1
64		**LepR**	Rn01433205_m1
65		**Npy**	Rn01410145_m1
66		**Mc4r**	Rn01491866_s1
67		**Pomc**	Rn00595020_m1
68	Endocannabinoïd System	**Cnr1**	Rn02758689_s1
69		**Mgll**	Rn00593297_m1
70		**TrpV1**	Rn00583117_m1
71		**Faah**	Rn00577086_m1
72	DNA methylation	**Dnmt1**	Rn00709664_m1
73		**Dnmt3a**	Rn01027162_g1
74		**Dnmt3b**	Rn01536418_g1
75	Histone deacetylases	***Sirt1***	Mm00490758_m1
76		**Sirt2**	Rn01457502_m1
77		**Hdac1**	Rn01519309_g1
78		**Hdac2**	Rn01193634_g1
79		**Hdac3**	Rn00584926_m1
80		**Hdac4**	Rn01427040_m1
81		**Hdac5**	Rn01464245_m1
82		**Hdac8**	Rn01419046_m1
83	Histone acetyl Transferases	**Kat6a**	Rn01211941_m1
84		**Kat5**	Rn01474465_m1
85		**Crebbp**	Rn00591291_m1
86	Serotonin	**Htr1a**	Rn00561409_s1
87		**Htr1b**	Rn01637747_s1
88		**Htr2a**	Rn00568473_m1
89		**Htr2c**	Rn00562748_m1
90		**Htr6**	Rn00577615_m1
91		**TPH2**	Rn00598017_m1
92		**Slc6a4**	Rn00564737_m1
93	House keeping genes	**Gapdh**	Rn01775763_g1
94		**Polr2a**	Rn01752026_m1
95		**Ppia**	Rn00690933_m1
96	Life Technologies Standar	**18S**	
