# Supplementary material for: Perinatal Western Diet Consumption Leads to Profound Plasticity and GABAergic Phenotype Changes within Hypothalamus and Reward Pathway from Birth to Sexual Maturity in Rat
Source: Front Endocrinol (Lausanne). 2017 Aug 29;8:216. doi: 10.3389/fendo.2017.00216 (PMC5581815; doi:10.3389/fendo.2017.00216)
Supplement: Table S2 — Summary of published data concerning dopamine pathway transcripts expression. The red characters correspond to childhood period, the blue ones to adolescence, and the black ones to adult. =: corresponds to a similar transcript expression between groups, +: corresponds to a higher transcript expression in pups from high caloric diet [junk food, western diet (WD), or high-fat diet (HFD)] fed dams, and −: corresponds to a lower transcript expression in pups from high caloric diet (junk food, WD or HFD) fed dams. [file table_2.pdf]

Supplementary Table 2: Summary of published data concerning DA pathway transcripts expression.

Strain/ Perinatal exposure	Postnatal exposure	Dopamine pathway qPCR analysis in male offspring															Behavior	References
		VTA					Nac					Hypothalamus						
		TH	DAT	D1	D2	MOR	TH	DAT	D1	D2	MOR	TH	DAT	D1	D2	MOR		
Rat SPD (Junk food 4W before mating still weaning)	Juvenile (adolescence) P42 (3 weeks junk food challenge)	-	--	=	=		=	--	=	=	++						Higher preference for fat (free choice)	21
	Adult P90 (junk food choice from weaning)	=	=	=	=		=	++	=	-	--						Higher preference for fat (free choice)	
Rat SPD (Junk food 4W before mating still weaning)	Juvenile (adolescence) P42 (3 weeks junk food challenge)	=	=	+	=	=			-	=	=						Higher preference for carbohydrate rather than fat (free choice)	35
	Adult P90 (Chow + 3weeks juvenile Junk food challenge)	=	=	+	=	=			=	=	=						No more difference (free choice)	
	Adult 6 months (chow + 3weeks young adult junk food challenge	=	=	=	-	=			=	+	=						No more difference (free choice)	
Rat SPD (WD from G1 to P21)	Childhood (P25) (chow from weaning)	=	=	=	=	=	=	=	=	++	=	=	=	=	=	=	Higher preference for fat (free choice)	this study
	Juvenile (adolescence) P45 (chow from weaning)	-	=	=	=	=	=	+	=	=	=	=	+	=	=	=	Less preference for fat in WD group	
	Adult P90 (chow from weaning)	=	=	=	=	=	-	=	=	=	+	=	+	-	=	=	No more difference	
Rat Wistar (WD from G1 to P21)	Adult P90 (chow from weaning)		=		=			=	=	=							No difference in motivation for high sugar reward (operant) but a higher preference for high palatable pellet (runway task)	22
Mice C57BL/6 (HFD 60% 3Month before mating)	Adult 18-24W (Chow from weaning)	=	++				-	++	--	--		++	--	=	=		Higher preference for fat	19
Rat SPD (HFD G13 to weaning)	Adult P90 (Chow from weaning)			=	-				=	=							Higher motivation for fat reward but not sugar	20
